# Methanol Synthesis from CO_2_: A Review of the Latest Developments in Heterogeneous Catalysis

**DOI:** 10.3390/ma12233902

**Published:** 2019-11-26

**Authors:** R. Guil-López, N. Mota, J. Llorente, E. Millán, B. Pawelec, J.L.G. Fierro, R. M. Navarro

**Affiliations:** Instituto de Catálisis y Petroleoquímica, CSIC, C/Marie Curie 2, Cantoblanco, 28049 Madrid, Spain; noelia.mota@icp.csic.es (N.M.); jorge.llorente@csic.es (J.L.); elena.millan.ordonez@csic.es (E.M.); bgarcia@icp.csic.es (B.P.); jlgfierro@icp.csic.es (J.L.G.F.)

**Keywords:** CO_2_, catalysts, hydrogenation, methanol, review

## Abstract

Technological approaches which enable the effective utilization of CO_2_ for manufacturing value-added chemicals and fuels can help to solve environmental problems derived from large CO_2_ emissions associated with the use of fossil fuels. One of the most interesting products that can be synthesized from CO_2_ is methanol, since it is an industrial commodity used in several chemical products and also an efficient transportation fuel. In this review, we highlight the recent advances in the development of heterogeneous catalysts and processes for the direct hydrogenation of CO_2_ to methanol. The main efforts focused on the improvement of conventional Cu/ZnO based catalysts and the development of new catalytic systems targeting the specific needs for CO_2_ to methanol reactions (unfavourable thermodynamics, production of high amount of water and high methanol selectivity under high or full CO_2_ conversion). Major studies on the development of active and selective catalysts based on thermodynamics, mechanisms, nano-synthesis and catalyst design (active phase, promoters, supports, etc.) are highlighted in this review. Finally, a summary concerning future perspectives on the research and development of efficient heterogeneous catalysts for methanol synthesis from CO_2_ will be presented.

## 1. Introduction

The current world energy system is still mainly based on the use of fossil fuels and, although the use of renewable energy sources has increased, it will continue in the medium and short term [1]. This massive use of fossil fuels in industry and transport produce large amounts of CO_2_ emissions [2] that could reach 35.2 billion metric tons in 2020 [3]. Therefore, it is necessary to develop technological approaches to reduce these CO_2_ emissions associated with the use of fossil fuels which must include the capture and subsequent reutilization of the CO_2_ produced [2]. In this scenario, to meet a climate target of limiting warming by 2 °C, annual energy-related CO_2_ emissions still need to decline by 2050 from 35 Gt (in the current levels) to 9.7 Gt, a decrease of more than 70% [1]. To reach this objective, the reutilization of 7–32% of the CO_2_ produced in the generation of energy from fossil fuels will be necessary by 2050 [1]. As an illustrative example, this means that the CO_2_ emissions in the energy sector in the EU-28 are expected to be reduced to 1550 Mt (mega ton) by 2030 from the 3400 Mt emitted in 2013 [4]. Therefore, technologies which enable the effective re-utilization of CO_2_ for manufacturing value-added compounds or fuel products will play a major role in the objectives related with the reduction of CO_2_ in the future.

Today, the main chemical products obtained at the industrial scale using CO_2_ as a raw material (pure or derived from CO by the Water Gas Shift reaction (WGS)) are urea, methanol, formaldehyde, methanol, formic acid, carbamates, polymer-building blocks and fine chemicals (Table 1) [5]. Among them, the synthesis of urea and methanol are the predominant consumers of CO_2_ in industry with an annual consumption of CO_2_ of more than 110 Mt/year.

One of the most interesting products that can be synthesized from CO_2_ is methanol [6]. Methanol is an industrial commodity used as a feedstock in several industrial chemicals and fuels products [2]. The main chemical methanol derivatives are formaldehyde, acetic acid, methyl tertiary-butyl ether (MTBE) and dimethyl ether (DME). The methanol transformation in olefins is an emerging sector [5,6]. The chemical versatility of methanol as well as the possibility to use liquid methanol with the existing infrastructures for fuel transportation and distribution is the basis of the so-called “*methanol economy*”, a concept proposed in 2005 by Olah et al. [7,8,9,10].

The global production of methanol has been growing from 2009 (53.9 Mt) to 2012 (58 Mt) which represents a growth of 7% according to the International Energy Agency (IEA) [11]. In the same way, the capacity of methanol production worldwide has also grown by 10% to reach 95.5 Mt in 2012 and 98.3 Mt in 2013. Europe represented 3% of world production [12,13], while China is the largest producer with 50% of the world’s capacity [14]. The largest consumer of methanol is the synthesis of formaldehyde (31% of methanol consumed in 2013) [15]. Methanol consumption in direct fuel applications is another of the main uses of methanol, and it includes methyl tertiary-butyl ether (MTBE), tert-amyl methyl ether (TAME) and dimethyl ether (DME), which together they account for 37% of the global methanol demand [15]. In the next years, a 10% increase is expected in the demand for methanol caused by the rise in formaldehyde demand at an average rate of 5% and by the growth of demand for it as a fuel which will increase at a rate of around 6.5% [14].

This review reports the most significant advances made in the heterogeneous catalytic hydrogenation of CO_2_ to methanol and the barriers that need to be addressed, low conversion and methanol selectivity, over the next coming years to convert this technology in a competitive process in the future systems of production and storage of energy and chemicals from renewable resources.

## 2. CO_2_ Activation and Processes for Its Hydrogenation into Methanol

The CO_2_ molecule is difficult to be activated due to the fact of its thermodynamic stability (Δ_f_*G*^0^ = −394.38 kJ mol^−1^) and kinetic inertness [5]. Carbon dioxide is a linear non-polar molecule with two reactive sites: oxygen and carbon. The electron deficiency of the carbonyl carbon means that CO_2_ has strong affinity towards nucleophiles and electron-donating reagents, while the oxygen atom shows an opposite behaviour [16]. Taking these characteristics into account, an external energy input and an efficient catalyst are prerequisite for converting CO_2_ into methanol, because the conversion of CO_2_ is kinetically limited [17]. Nowadays, different process approaches are being developed for the synthesis of methanol by hydrogenation of CO_2_: (1) heterogeneous catalysis, (2), homogeneous catalysis, (3) electrochemical, and (4) photocatalysis.

### 2.1. Heterogeneous Catalysis

The process for synthesis of methanol from syngas was developed in the 1940s [18] and became widespread in the 1960s [19]. This process is based on the use of Cu–ZnO heterogeneous catalysts, in which Cu is the active phase and ZnO is the essential promoter to improve the activity of the system [20]. Conventional process for the exothermic CO_2_ hydrogenation into methanol (ΔH 298 K = −49.5 kJ mol^−1^) involves the catalytic conversion at low operation temperatures (230–270 °C) with several stages due to the kinetic limitations (15–25%) [17]. Carbon dioxide is a very stable molecule, and conventional syngas Cu–ZnO catalysts could be applied, but high deactivation and low activities are observed respect its use with syngas. Therefore, great efforts were made in the 1990s for the development of catalysts in order to improve the low activity and the high degree of deactivation of the Cu–ZnO catalysts. The result was the development of new catalysts and processes [21]. This brief review focuses on the most recent advances in heterogeneous catalytic hydrogenation of CO_2_ to obtain methanol. Particular attention is given to the literature that reports the latest efficiency improvements in conventional Cu–ZnO catalysts and the new strategies to develop innovative alternative catalysts with non-Cu as active phase.

### 2.2. Homogeneous Catalysis

Most of the developments in homogeneous catalysts for the hydrogenation of CO_2_ are focusing on the synthesis of formaldehyde or formic acid [22]. The first study in the hydrogenation of CO_2_ to produce methanol using homogeneous catalysts was published by Tominaga et al. [23] in 1995. In general, ruthenium complexes with several ligands have been the most studied homogeneous catalyst for this process [24,25] among which Ru-Triphos (Triphos = 1,1,1-tris (diphenylphosphinomethyl) ethane) has been identified as one of the most effective [26]. The hydrogenation of CO_2_ using a molecularly defined single-site catalyst allows clarifying the reaction steps and mechanism which the reaction passes. Initially, it was suggested that the reaction takes place with methyl formate as an intermediate [27], but, currently, it is accepted that the transformation occurs directly in the homogeneous catalyst in several steps in which the cationic format complex ((Triphos) Ru (η2-O_2_CH) (S)) + (S = solvent) has been identified as the key intermediate. The mechanism is composed of a sequential series of hydride transfer and protonolysis steps which transform the CO_2_ into formate/formic acid, hydroxymethanolate/formaldehyde and finally methanolate/methanol within the sphere of coordination of a single Ru-Tripho fragment [26]. In addition to this ligand, ligands that include three-membered heterocycles containing oxygen, nitrogen, or unsaturated compounds, and other reducing reagents could be favourable to react with CO_2_ and promote the formation of bonds: C–O/C–N/C–C. Thus, derivatives of urea, cyclic carbonates, polycarbonates, acetylsalicylic acid and salicylic acid are also complexes that could be interesting candidates to use in the homogeneous hydrogenation of CO_2_ for the synthesis of methanol.

### 2.3. Electrochemical

The electrochemical activation of CO_2_ by electrocatalysts allows the hydrogenation to methanol under mild conditions. Metals as Pt [28,29], Pd [29], and Ru [30] have been studied as catalysts in the electrochemical activation of CO_2_, in general supported on Na- or K-modified β-alumina, in order to increase the conductivity of the ceramic β-alumina and the chemisorption of CO_2_ and H_2_ over the metal active sites [29,30]. Non-expensive metals, such as Cu supported on K-β-alumina [31] or Ni supported on YS zeolite [32], have also been studied. Molecular organometallic electrocatalysts have also demonstrated activity and selectivity for the electrocatalytic reduction of CO_2_. An effective approach to improve the activity and efficiency of these molecular electrocatalysts is the modulation of the secondary coordination sphere of the active sites [33]. This has been achieved by mimicking enzymatic structures incorporating of Brønsted acid/base sites, charged residues and sterically hindered environments (Fe(tetraphenylporphyrin))^+^, (Ni(cyclam))^2+^, Mn(bpy)(CO)_3_X, and Re(bpy)(CO)_3_X (X = solvent or halide). High-quality review articles on electrochemical methods for CO_2_ reduction are available for readers interested in this area [34].

### 2.4. Photocatalysis

The basis of this technology is the design of efficient photocatalysts for harvesting solar energy [35] and to perform the hydrogenation of CO_2_ [36]. Different photocatalysts have been studied in the photoreduction of CO_2_ to methanol. Conventional photocatalysts based on titanium dioxide (TiO_2_) nanoparticles supported on the surface of reduced graphene oxide were used as photocatalyst with high activity in methanol synthesis from CO_2_ reduction (methanol yield: 2330 μmol g_cat_^−1^ h^−1^) [37]. The photoactivity of TiO_2_ under solar illumination is low due to the limited portion of UV radiation in the total spectre [38]. Nevertheless, the nanocomposite TiO_2_-reduced graphene allows decreasing the band gap of TiO_2_ due to the contribution of reduced graphene oxide. Other type of photocatalysts tested for this process are based on the cuprous and cupric oxides (Cu_2_O and CuO) supported on the surface of the reduced graphene oxide [39]. Among them, the Cu_2_O with rhombic dodecahedral structure exhibits the best photocatalytic activity of CO_2_ reduction with values of methanol yield of 355.3 μmol g_cat_^−1^. Another approach is the use of CuO–ZnO photocatalysts in hierarchical heterojunction in the form of nanospheres that allow the effective photoreduction of CO_2_ to methanol (3855.4 μmol g_cat_^−1^) under visible light. This type of CuO/ZnO heterojunction allows an improved electron–hole pair separation which reduces its recombination [40]. Another example of photocatalysts for CO_2_ reduction to methanol is the synthesis of nanostructured materials with polymeric carbon nitrides C_3_N_4_ nanosheets in combination with CdSe quantum dots. Based on the quantum confinement effects of CdSe quantum dots, the energy of the electrons can be adjusted to a level that improves the selectivity and activity for methanol production [41]. Another active photocatalyst used in the hydrogenation of CO_2_ to methanol is that based on nanostructured MoS_2_ in form of nanosheets. The morphology of MoS_2_ in the form of two-dimensional structures allows for improving the mobility of the photogenerated electrons, which seems to be the key to obtaining methanol yield of 109.5 μmol·g_cat_^−1^ [42]. Despite the advances in the development of photocatalysts during the last years, these systems show rather low efficiency for methanol production and several issues have to be addressed to achieve its practical application: (i) the charge recombination; (ii) an unsatisfying stability and (iii) poor selectivity. Readers interested in the approaches and opportunities of CO_2_ reduction driven by solar energy using both material design and reactor engineering are pointed to some excellent reviews published in this area [43,44].

## 3. Heterogeneous Catalytic Hydrogenation of CO_2_ to Methanol

### 3.1. Differences and Requirements in Respect to Conventional Synthesis from Syngas

Methanol is currently produced at the industrial scale from synthesis gas (syngas), with a CO/H_2_ composition called *metgas*, according to the stoichiometric Equation (1). The *metgas* is predominantly derived from the steam reforming of fossil fuels (mainly methane) and it contains small concentration (3 vol.%) of CO_2_ [45]. The *metgas* stream passes over conventional copper-based (Cu/ZnO/Al_2_O_3_) catalysts at high pressure (5–10 MPa) and moderate temperature (200–300 °C).

CO + 2 H_2_ ⇌ CH_3_OH ΔH = −90.8 kJ mol^−1^ (1)

The synthesis of methanol from CO_2_ (Equation (2)) is less exothermic than that starting from syngas, and it also involves as secondary reaction the reverse water–gas-shift (RWGS) (Equation (3)) [46].

CO_2_ + 3 H_2_ ⇌ CH_3_OH + H_2_O ΔH_298K_ = −49.5 kJ mol^−1^ (2)

CO_2_ + H_2_ ⇌ CO + H_2_O ΔH_298K_ = 41.2 kJ mol^−1^ (3)

Previous studies describing the mechanism of methanol synthesis from syngas proposed that methanol is most probably formed by hydrogenation of CO_2_ contained in syngas and, therefore, the catalysts and catalytic steps on catalyst surfaces are the same in the hydrogenation of syngas or CO_2_. However, the main differences in the synthesis of methanol from pure CO_2_ or syngas are derived from the differences in the exothermicity of both processes and from the higher production of water formed during the synthesis from CO_2_ which deactivates prematurely the catalysts [47]. Formation of methanol from syngas is a process highly exothermic (Equation (1)). Therefore, the main priority for the reactor design is removal of the excess of heat generated during the reaction. Thus, the boiling water reactor type is the reactor used in conventional plants of methanol synthesis from syngas, since this reactor type facilitates the dissipation of the heat generated. Conversely, the thermal control inside the reactor during the methanol synthesis from CO_2_ is easier due to the lower heat profile of this process. In this case, boiling water reactor is not required. A simple tube-cooled reactor is sufficient to control the temperature in the synthesis of methanol from CO_2_ which makes it possible to lower the cost and the efficiency of operation [46].

Conventional catalysts for methanol synthesis from syngas are based on Cu [18,45] in combination with ZnO [20,48,49,50,51,52,53]. Nevertheless, these catalysts are less effective in the synthesis of methanol from CO_2_-rich feeds [48]. Actually, the CO_2_ conversion remain low (<20%) due to the difficulties in activation of the CO_2_ molecule [49] and because it is a kinetically limited (15–20%) process [50]. The higher production of water formed during the synthesis from CO_2_ is also an issue that should be addressed because the excess of water can accelerate the crystallization of Cu and ZnO particles in the catalysts which result in rapid sintering and deactivation together with the formation of other unwanted by-products such as higher alcohols or hydrocarbons [54]. Nevertheless, the most commonly used and studied catalysts for the selective hydrogenation of CO_2_ to methanol are those Cu–ZnO-based catalysts [45]. Therefore, improvements in the formulation, morphology and physicochemical properties of catalysts for CO_2_ hydrogenation must be developed in order to obtain new efficient catalysts with greater activity and methanol selectivity than those which now exist.

### 3.2. Conventional Cu–ZnO–Al*_2_*O*_3_* Catalysts

The active sites in conventional Cu–ZnO catalysts are related with partial or completely reduced Cu [45,47] with a synergic contact with ZnO or partially reduced ZnO_x_ [47,50,55,56,57]. The development of Cu–ZnO contacts is decisive for the activity, although there is no unambiguous explanation for its role and several options are proposed: (i) the ZnO favours the dispersion of reduced Cu (increasing the number of active sites), (ii) the active Cu^+^ sites were stabilized on the surface of the ZnO, (iii) the ZnO favours the Cu^2+^ reducibility, or (iv) the basic sites of ZnO in close contact with Cu-metal sites are necessary to catalyse the hydrogenation of carbon oxides [45,47,53]. Therefore, the formation of Cu–ZnO interphases can be a key factor to obtaining highly active catalysts for methanol synthesis from CO_2_ [53,55]. These interphases are formed during the reduction of the catalyst precursors in which an optimization of the reduction variables (temperature, heating rate, hydrogen partial pressure) play a decisive role for obtain catalysts with optimal activity [55]. Not only the nature of the Cu–ZnO interphase but also the exposed faces of the ZnO in contact with Cu species influence the catalytic behaviour of the Cu/ZnO systems [51,52]. Lei et al. [51] synthesized ZnO with different nanomorphology and they verified that the (002) face of the ZnO presented the best catalytic results in the synthesis of methanol since this face was more polar because it presents higher concentration of oxygen vacancies [51].

**Figure 1:**
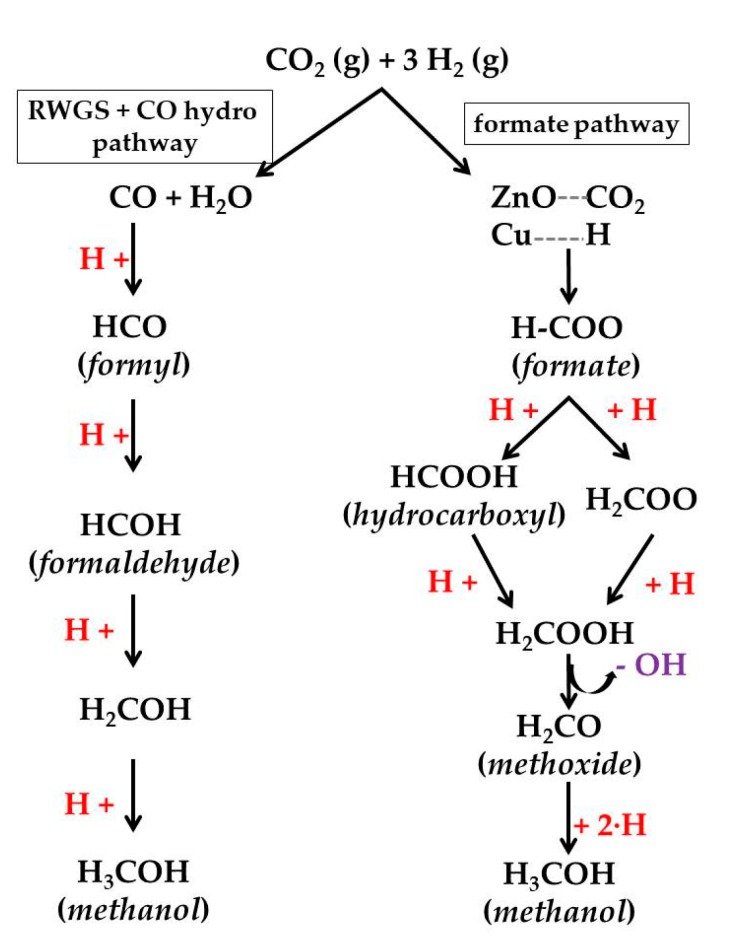
Pathways of methanol synthesis from CO_2_ hydrogenation over Cu-based catalysts (adapted from References [58,59]).

On the other hand, the understanding of the reaction mechanism operating on Cu/ZnO catalysts will also allow the improvement of the catalysts for this process. In this sense, there are two mechanisms that are mostly accepted for the hydrogenation of CO_2_ on Cu/ZnO catalysts (Figure 1) [58,59]: (a) formate mechanism, in which CO_2_ hydrogenation produces formate intermediates (HCOO), and a (b) reverse water–gas-shift (RWGS) and CO hydrogenation mechanism, where CO_2_ is converted to CO, followed by CO hydrogenation to methanol via formyl (HCO) and formaldehyde (HCHO) intermediates [47]. The intermediate product common in both mechanisms, formaldehyde, can be hydrogenated to methoxy and from them the final product, methanol, can be obtained [17,45,47,49,56]. Kinetic studies have identified the hydrogenation of formate (HCOO) and dioxomethylene (H_2_COO) species as the limiting steps for the methanol synthesis via the formate pathway [17,47,51]. On the other hand, the alternative pathway of RWGS and hydrogenation of CO has as a limiting step of the hydrogenation of CO and formyl (HCO). Thus, it appears that the methanol yield in the hydrogenation of CO_2_ carried out with Cu/ZnO catalysts is controlled by three factors: (i) the dioxomethylene hydrogenation barrier, (ii) the CO binding energy and (iii) the CO hydrogenation barrier. An ideal Cu-based catalyst for methanol synthesis via CO_2_ hydrogenation should be able to hydrogenate dioxomethylene easily and bond CO moderately, being strong enough to favour the desired CO hydrogenation rather than CO desorption but weak enough to prevent CO poisoning. In this way, the methanol production via both the formate and the RWGS with CO hydrogenation pathways can be facilitated [17]. The knowledge of which of these two mechanisms occur in the synthesis of methanol is a decisive criterion for the design of the catalysts for the hydrogenation of CO_2_.The methanol is mainly produced via the formate route [51,60] since, although the reaction rate of hydrogenation of CO_2_ by RWGS is faster than the rate of hydrogenation of CO_2_ by the formate pathway, the presence of CO_2_ decreases the reaction rate of methanol synthesis from syngas [61]. Consequently, the design of new Cu–ZnO catalysts should improve the suppression of the active sites for RWGS and the robustness of the methanol synthesis sites against the inhibition of the water product. In the Cu–ZnO catalysts, Cu-sites are responsible for the adsorption and dissociation of the H_2_, while the ZnO was responsible for the adsorption of CO_2_ as bicarbonate species. Therefore, the methanol synthesis on Cu–ZnO catalysts in accordance with the formate mechanism passes through the following steps: (1) formate species formed on Cu–ZnO contacts [51] by reaction of CO_2_ adsorbed on ZnO with the surface atomic H formed on Cu sites [57]; (2) the formate species are hydrogenated to methoxy which are adsorbed on the ZnO; and (3) methoxy is hydrolysed to methanol [51,57,61]. Nevertheless the last step depends on the amount of oxygen and water on ZnO (ZnO/ZnO_x_) in a such a way that the presence of adsorbed water suggests that methanol is produced through a carboxyl intermediate [53], because the hydrogenation route to hydrocarboxyl (Figure 1) is kinetically more favourable due to the hydrogen transfer mechanism [58,61].

## 4. Advances in Heterogeneous Catalysts for Methanol Synthesis from CO_2_

### 4.1. Modifications for Conventional Cu–ZnO–Al_2_O_3_-Based Catalysts

#### 4.1.1. Improvements in Synthesis

As indicated previously, the state-of-the art catalyst for the synthesis of methanol from CO_2_ is the Cu/ZnO/Al_2_O_3_ formulation. In an analogy to the synthesis of methanol from syngas [53], the active sites for CO_2_ hydrogenation in conventional Cu-based catalysts are related with partial or completely reduced Cu with a synergic contact with ZnO or partially reduced ZnO_x_ [61]. The ZnO plays an important role in the performance of the catalysts, because it favours the dispersion and stabilization of Cu active sites and also facilitates the adsorption of CO_2_ to be subsequently hydrogenated to methanol [56]. In the case of Al_2_O_3_, it improves the exposition and stabilization under reaction conditions of the Cu-active centres. The conventional catalysts for synthesis of methanol have proven to be structure sensitive, because its activity can vary by orders of magnitude, depending on how it is prepared [21]. The conventional preparation methodology for the Cu/ZnO/Al_2_O_3_ catalyst is co-precipitation which includes three stages: (i) the precipitation of the precursors in the form of hydroxycarbonates [62,63,64], (ii) the controlled calcination of these hydroxycarbonate precursors to produce highly dispersed CuO–ZnO species with some residual carbonates which are responsible for maintaining high porosity and surface area, and (iii) the reduction of these oxidized phases to obtain the active catalyst as Cu^0^ or Cu^+^ nanoparticles decorating the phases of ZnO or partially reduced ZnO_x_ [62,63].

**Figure 2:**
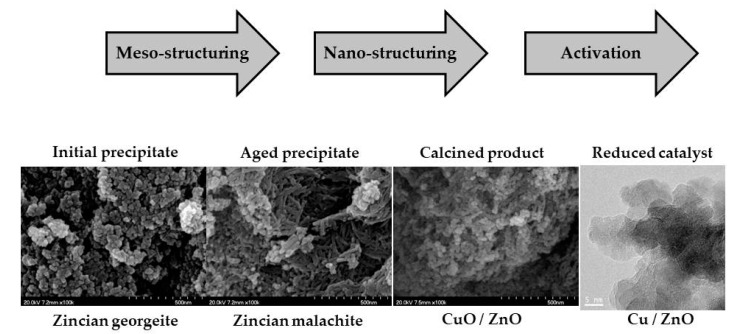
Microscopy images of the materials obtained at different stages in the course of the preparation of Cu/ZnO/Al_2_O_3_ catalysts.

The synthesis of the hydroxycarbonate precursor is a key stage of the catalyst synthesis, because the activity of the final catalyst strongly depends on the properties fixed during these early stages of the catalyst preparation [19,64]. Therefore, the control of the precipitation of catalyst precursors is essential for the preparation of a good catalyst for the hydrogenation of CO_2_. The desired properties in the final catalysts are a high Cu surface area (depending on small particle size and meso-porosity), good Cu–ZnO interaction or even formation of surface Cu–Zn partial oxidized mixed oxides, and minimal contamination from alkali metals (Na^+^) introduced during the co-precipitation process [65]. The selective preparation of the hydroxycarbonate precursors involves the control of parameters in the precursor formation as elemental formulation pH, temperature, ageing time [63,64], and complete removal of Na^+^ [63,65,66]. There is some controversy in the literature about which of the hydroxycarbonate phases is the best to obtain an optimal catalyst for the synthesis of methanol from CO_2_. In this way, different studies have been carried out with catalysts derived from aurichalcite ((Cu,Zn)_5_(CO_3_)_2_(OH)_6_) [62,67], zincian malachite ((Cu,Zn)_2_CO_3_(OH)_2_) [65,68,69], hydrotalcite (Cu_1−x−y_ZnyAlx(OH)_2_(CO_3_)_x/2_) [70,71], and even the amorphous zincian georgeite phase ((Cu,Zn)(CO_3_)(OH)_2_) [65,69,72]. The best catalytic performance seems to be linked with the presence of the zincian malachite phase in the hydroxycarbonate precursor with a maximum amount of Zn incorporated into the structure of the malachite [57,58,59,60,61,62,63,64,65,66,67,68,69,70,71,72]. However, recent studies also indicate that there is an additional factor to the presence of zincian malachite that is also key to obtaining a good catalyst for the synthesis of methanol. In this sense, the presence of amorphous zincian georgeite has also been highlighted as a cooperative phase for improving the catalytic behaviour over the single phase of malachite zincian [63,65,69,70,71,72]. During the precipitation, the hydroxycarbonate precursors evolve in the preparation medium. It passes first through an amorphous stage (georgeite) that evolves into malachite. It is at this stage that the mesostructure of the final catalyst is defined while the nano-structuring of the catalysts is defined after the calcination step (Figure 2). The amorphous zincian georgeite phase was responsible of the maintenance of the nano-structuring of the catalysts after the calcination of hydroxycarbonate precursors because it maintains the carbonate structure after calcination (high temperature carbonates). These carbonates remaining after calcination allow to obtain small CuO–ZnO nano-domains in good contact which guarantee a low reduction temperature of CuO and small particle size of the metallic Cu with good contact with ZnO/ZnO_x_ interfaces after reduction [62,63,73].

On the other hand, the reduction conditions of the calcined precursors must also be controlled to avoid the sintering of the Cu particles and to control the amount of Cu^+^ in the catalysts after reduction. Some authors postulate that the presence of high amounts of Cu^+^ species is crucial to reach high methanol selectivity [74]. Therefore, the Cu^+^/Cu^0^ ratio and its distribution are variables to tune in the preparation and design of optimized catalysts for the selective CO_2_ hydrogenation to methanol.

#### 4.1.2. Promoters

The addition of promoters to the conventional Cu/ZnO/Al_2_O_3_ catalysts for CO_2_ hydrogenation to methanol has also been well studied. Different types of promoters have been studied to modify the Cu active sites or the physicochemical characteristics of the catalysts (basicity, reducibility) by interaction with the Al_2_O_3_ component. Regarding the addition of promoters to modify the Cu active sites, most of the studies focused on noble metals for the formation of Cu–Me alloy phases [17,50,53,61,75]. The addition of noble metals such as Pt, Rh, Au or Pd increases the activity of Cu catalysts during the hydrogenation of CO_2_. Nevertheless, on the one hand, the increase in the performance of these catalysts based on noble metals with respect to that obtained with conventional Cu catalysts does not justify the price increase associated with the use of noble metals. On the other hand, at present, there are no studies focused on the recovery of these noble metals once they are exhausted after reaction. Therefore, their industrial use is not advised at present.

Other studies about the modification of conventional Cu–Zn–Al catalysts with promoters focus on the modification of the basicity and physicochemical properties of the catalyst through the interaction of promoters with alumina [50]. These studies mainly focus on the use of three promoters: zirconium [50,54,64,76], gallium and fluoride [50,73,74,77]. Zirconium (Zr) as promoter acts at three levels: (i) it modifies the formation of the hydroxycarbonate precursors during precipitation because it favours the formation of hydrotalcite precursors; (ii) the presence of ZrO_2_ stabilizes the Cu^+^ species in reducing environments avoiding its deactivation [53,54]; and (iii) the addition of Zr increases the basicity of the catalyst. The effect of Zr as promoter on Cu–Zn–Al catalysts depends on its concentration (Table 2) [76], for Al/Zr ratio up to 2.33 the dispersion of Cu and conversion of CO_2_ increases while for higher Al/Zr ratio both values decreases.

The studies about the promoter effect of Ga on the performance of the Cu–Zn–Al catalysts suggest that the concentration of Cu^0^/Cu^+^ species could be regulated by varying the Ga content in the catalysts modifying by this way their catalytic performance [56]. It was shown that Ga is incorporated into the zincian malachite structure during precipitation [66]; moreover, the co-loading with Al^3+^ and Ga^3+^ increases with the insertion of Ga into malachite precipitates respect to the Al- or Ga-mono-substituted counterparts. The activities of these catalysts (Table 3) suggest a different participation of the copper surfaces in combination with the partially reduced ZnO_x_ sites depending on the load of Al and Ga. The sample with an Al/Ga = 1 ratio shows a marked improvement in activity with respect to the Al- or Ga-mono-substituted counterparts. This improvement in activity seems to be related to changes in ZnO defects with the substitution Zn^2+^/Al^3+^/Ga^3+^ which was achieved in the sample with the same amount of Ga and Al that produced a surface contact different between Cu and reduced ZnO which resulted in sites with higher activity for methanol synthesis [66].

The addition of fluoride ions as promoters of the Cu–ZnO–Al_2_O_3_ catalysts was also studied as a way to decrease the acidity of the alumina species [77]. The incorporation of fluoride ions is achieved during precipitation of precursors because the hydroxycarbonates in the form of hydrotalcites allow the incorporation and stabilization of small amounts of fluoride ions in its structures. The promotional effect associated to the presence of fluoride ions was related to the increase in the adsorption of CO_2_ which favours, as commented previously, their selective hydrogenation into methanol.

### 4.2. Cu–ZnO Supported Catalysts

Other modifications of the Cu–ZnO catalyst explore the total substitution of the aluminium from the catalyst formulation using supports based on other tri- or tetravalent metal oxides. One of the most studied oxides to combine with Cu–ZnO is zirconium oxide, ZrO_2_ [53,61,78]. Zirconium oxide has a weak hydrophilic character in comparison to alumina, which could enhance the copper dispersion and stability [78] impeding the absorption of water. Moreover, the substitution of Al_2_O_3_ by ZrO_2_ increases the basicity of the final catalyst [78] which favours the selectivity to methanol due to the higher CO_2_ adsorption over the basic sites and its subsequent hydrogenation on the Cu–ZnO active sites [47,79]. Comparative studies between alumina, Al_2_O_3_, ZrO_2_ [78] or CeO_2_ [80,81] as modifiers of the Cu/ZnO sites have proven the best catalytic behaviour in the sample modified with ZrO_2_ (Table 4) [81].

**Figure 3:**
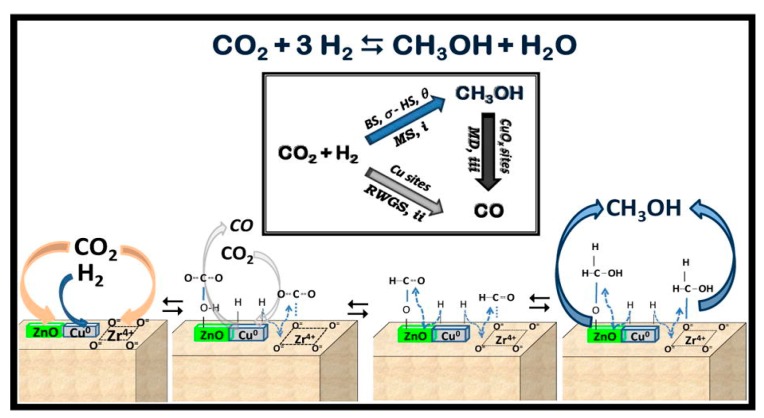
Reaction network on Cu–ZnO/ZrO_2_ catalysts [79].

The ZrO_2_ favours the formation of oxygen vacancies on the surface during reduction and high Cu dispersion because the microcrystalline copper particles are stabilized by interaction with zirconia [80]. The enhanced reactivity of ZrO_2_-promoted Cu catalysts is also associated with changes in the functionality of Cu sites [79,80,81]. In this sense, as shown in Figure 3, the origin of the superior promotion effect of ZrO_2_ seems to be associated with the fine tuning capacity of reduced Zr^3+^ species at the Cu/ZnO interface, being able to bind the key reaction intermediates, e.g., *CO_2_, *CO, *HCO, and *H_2_CO, in a moderate way to facilitate the formation of methanol [82].

Another element studied to substitute Al_2_O_3_ in Cu/ZnO catalysts is gallium oxide (Ga_2_O_3_). The presence of a small amount of Ga^3+^ in Cu/ZnO systems can facilitate the thermal reduction of ZnO and, consequently, highly active Cu–ZnO_x_ nanoparticles can be generated. This is achieved through the formation of gallium spinel, ZnGa_2_O_4_, which creates an electronic heterojunction with ZnO which facilitates its reduction and the formation of Cu–ZnO_x_ nanoparticles [83]. The increase in the concentration of Zn^0^ at the interface with metallic Cu particles can improve the catalytic performance of the methanol synthesis reaction from the hydrogenation of CO_2_. The formation of the type II heterojunction between ZnGa_2_O_4_ and ZnO also participates in the improvement in the methanol synthesis from CO_2_ because the heterojunction can modify the adsorption strength of the surface intermediates of HCO, H_2_CO, and H_3_CO facilitating their transformation into methanol [83].

Another effect of Ga^3+^ is associated with modifications during the synthesis of the hydroxycarbonate precursors, because the presence of Ga^3+^ facilitates the formation of precursors with hydrotalcite structure [84]. Catalysts derived from hydrotalcite precursors modified with Ga show improvement in the Cu dispersion and the formation of active Cu–ZnO_x_ sites which makes the Cu–ZnGa-containing catalyst a suitable candidate material for CO_2_ hydrogenation to methanol, even better than those derived from conventional hydroxycarbonate phases [84]. This happens because catalysts derived from hydrotalcite structures can maintain their morphology in ultrafine layers despite exhibiting an amorphous phase after calcination. These amorphous phases involve accessible, well-dispersed, small, large-area metal Cu crystals decorated with a trace amount of Zn atoms [84].

Mesoporous materials, as SBA-15, of high specific surface area which favour the dispersion of the active phase Cu/ZnO have been also studied as supports for this purpose. The supporting of Cu/ZnO on SBA-15 [85,86] or on SBA-15 modified with ZrO_2_ [85] has been studied. The catalytic behaviour of these materials indicated that the confinement of the Cu/ZnO particles in the SBA-15 structure improves its ability to interact with H_2_ and CO_2_ which results in better performance of the catalysts. Moreover, the confinement of the Cu/ZnO particles stabilizes them and allows for obtaining an optimum inter-particle spacing combined with a uniform distribution which stabilize the Cu/ZnO-SBA-15 catalysts compared to the conventional Cu/ZnO/Al_2_O_3_ [86]. The studies also show that the catalytic behaviour of Cu/ZnO supported on SBA-15 depends on both the Cu/ZnO loading and the Cu/Zn molar ratio because it leads to differences in the morphology and dispersion of the Cu/ZnO particles in the SBA-15 structure. The best results were obtained when a thin homogeneous amorphous layer of the Cu/ZnO particles formed inside the channels, instead of elongated particles with a diameter close to those of the mesopores or larger particles located on the external surface. This optimum morphology leads to an improvement in the interactions among the different Cu/ZnO particles which, in turn, determine an improvement in terms of catalyst activity and selectivity [85].

Carbonaceous materials have also been studied as supports for the Cu/ZnO particles. Different carbon morphologies from carbon nanotubes (CNTs) [87], graphene oxides aerogels [88], or various bimetallic Cu–Zn polymeric materials (BTC) [89] are found in the literature. The Cu/ZnO particles supported on CNTs presented nanoparticles of the active phase deposited both inside the CNTs as well as in the outer walls of the CNTs. The smaller particle size obtained with the CNTs support as well as the lower interaction of the active phase with the carbonaceous support in respect to the conventional alumina resulted in a greater reducibility of the catalyst. However, these active sites supported on CNTs gave methyl formate as a main product [87]. The use of reduced graphene oxide aerogel as support for Cu/ZnO particles in the direct hydrogenation of CO_2_ to methanol provided high surface area (458 m^2^ g^−1^) and achieved a high methanol production (2950.4 μmol CH_3_OH g_cat_^−1^ h^−1^ at 250 °C and 1.5 MPa). The bimetallic coordination polymer CuZn–BTC was studied as a support for the active phase Cu/ZnO for the methanol synthesis from CO_2_ and this support allows to obtain bimetallic ions evenly distributed, prevents the aggregation of Cu and ZnO nanoparticles and generates a great number stable Cu–ZnO interfacial sites which allows to maintain the selectivity to methanol at high temperature [89].

### 4.3. Other Cu-Based Catalysts

**Figure 4:**
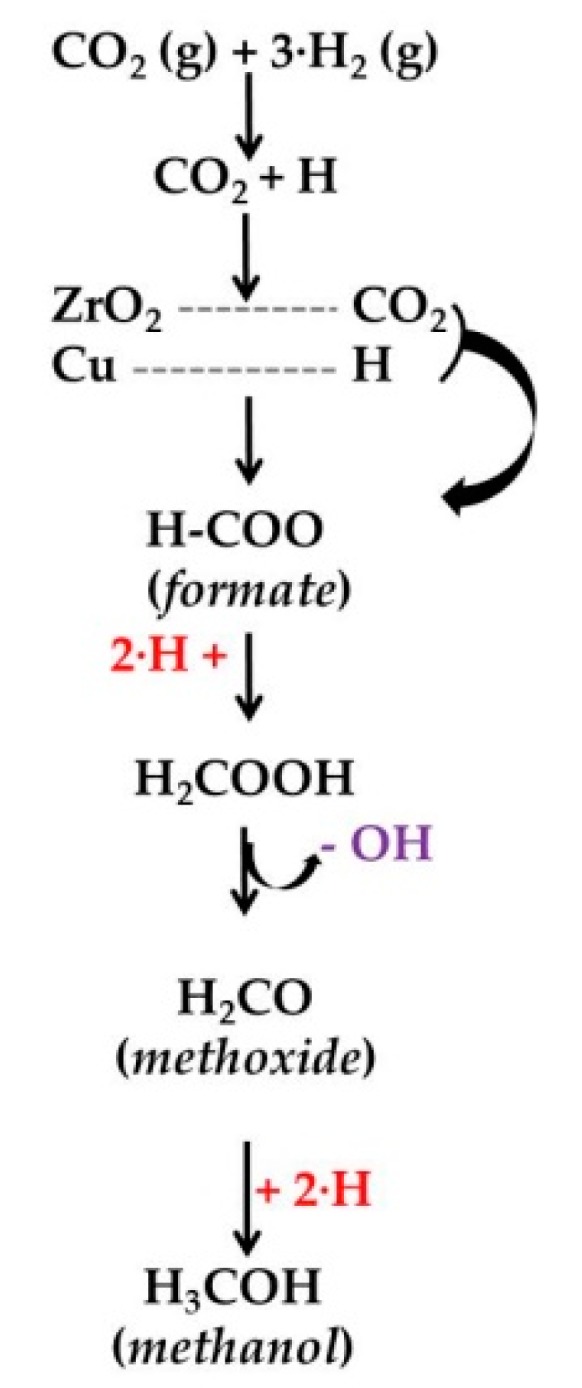
Reaction network to methanol on Cu/ZrO_2_ catalysts (adapted from Reference [90]).

In addition to the Cu–ZnO system, combinations of Cu nanoparticles with other oxides are also explored as a way to develop more effective and stable catalysts for the production of methanol from CO_2_. ZrO_2_ is a particularly promising oxide to combine with Cu because it leads to a highly active, selective, and stable catalysts. According to a recent study [90], the mechanism for the hydrogenation of CO_2_ to methanol on Cu/ZrO_2_ catalysts involves the adsorption of CO_2_ on ZrO_2_, forming formate species with the concomitant dissociative H_2_ adsorption on the surface of Cu [90] and the transference of the H atoms to the formate species on ZrO_2_ which transform into methoxy species (Figure 4). In a subsequent step, the methoxy species are hydrogenated to methanol product [90].

**Figure 5:**
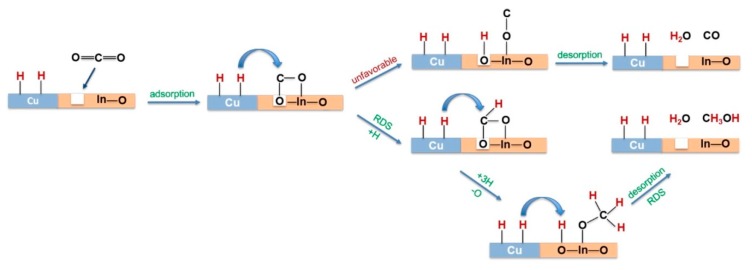
Representation of the bifunctional Cu and defective In_2_O_3_ sites operating in methanol synthesis from CO_2_ on Cu/ZrO_2_–In_2_O_3_ catalysts [92].

The Cu/ZrO_2_ catalysts have also been modified with Ag or In_2_O_3_ as a way to improve its efficiency. The addition of Ag to the Cu/ZrO_2_ catalysts produces modifications in the surface area, the concentration of partially reduced ZnO_x_ sites, and the formation of Ag–Cu alloy [91]. The presence of Ag^+^ in the CuO–ZrO_2_ catalysts decreases its specific surface area and its meso-structuration but increases the amount of partially reduced ZrO_x_ sites. In reduced Ag–Cu/ZrO_2_ catalysts, it was observed the formation of Ag–Cu alloy which shows a higher methanol production rate (7.5 mL g_cat_^−1^ h^−1^, CO_2_/H_2_/N_2_ = 1/3/1, W/F_total_ = 1000 mg_cat_ mL^−1^ s^−1^, *Tr* = 230 °C, *P* = 1.0 MPa) compared to the reduced CuO/ZrO_2_ counterpart (6.9 mL g_cat_^−1^ h^−1^) [91]. Modification of the ZrO_2_ support with indium oxide (In_2_O_3_) is also other alternative to improve the activity of the Cu/ZrO_2_ catalysts [92]. In this case, the catalysts are based on Cu–In–Zr–O mixed oxide containing bifunctional Cu and defective In_2_O_3_ active sites which promote the kinetics and selectivity in the hydrogenation of CO_2_ to methanol through a formate–methoxy–methanol route. The high methanol selectivity values are derived from the strong adsorption of CO_2_ on the In_2_O_3_ defects, which creates an energy barrier that suppresses the dissociation of CO_2_ into CO. The promotion in the kinetics of hydrogenation of CO_2_ was attributed to the cooperation between the Cu sites adsorbing and providing active hydrogen atoms that hydrogenate the CO_2_ adsorbed in the adjacent defective In_2_O_3_ sites (Figure 5).

The improvement of the activity of Cu/ZrO_2_ catalysts was also studied supporting them on mesoporous SBA-15 [93] or N-activated carbon nanotubes [94]. When using SBA-15 as a support, it is important to define the silica precursor, since the morphology of the SBA-15 changes and the incorporation of Cu occurs in different ways and at different sites. Thus, the Cu/ZrO_2_ grown into the pores of the SBA-15 support caused a decrease in activity derived from the decrease in the specific surface area and the size of the pores, while the homogeneous distribution of Cu/ZrO_2_ particles on the walls of the pores of SBA-15 in samples prepared with TEOS [93] causes an improvement in the catalytic activity. The Cu/ZrO_2_ catalysts supported on carbon nanotubes doped with N (CNTs-N) exhibit high activity and selectivity in the methanol synthesis from CO_2_ (3190.0 μmol CH_3_OH g_cat_^−1^ h^−1^ with 10 wt.% Cu loading at 3.0 MPa, 260 °C, V(H_2_):V(CO_2_):V(N_2_) = 69:23:8 and GHSV = 3600 mL g_cat_^−1^ h^−1^) [94]. The high activity of the Cu/ZrO_2_ supported on CNTs-N is related with the nitrogen species existing on carbon nanotubes, mainly pyridinic-N which interacts with Cu species improving its dispersion and promoting its reduction which leads to a smaller Cu crystal size and a high intrinsic activity. However, the conversion of CO_2_ is controlled predominantly by the adsorption of activated CO_2_. In this sense, the pyridinic-N also stimulates the strong absorption of activated CO_2_, contributing to the formation of methanol. On the contrary, pirrolic-N promotes the formation of CO by RWGS reaction [94].

**Figure 6:**
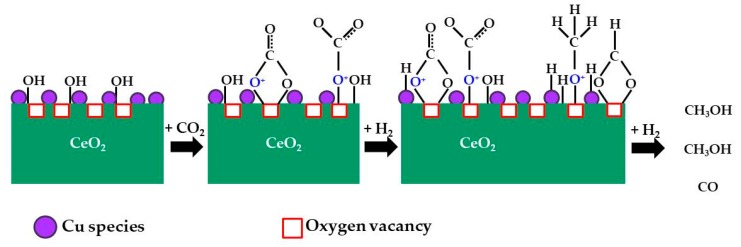
Intermediate species of CO_2_ adsorption and hydrogenation on Cu/CeO_2_ catalysts (adapted from Reference [95]).

Cerium oxide (CeO_2_) was also studied as an oxide to combine with Cu to obtain effective catalysts for the hydrogenation of CO_2_ to methanol [95]. The results of the methanol synthesis from CO_2_ at 3.0 MPa and temperatures between 200 and 300 °C revealed that the Cu catalysts supported on CeO_2_ exhibit better activity and selectivity than the monometallic Cu catalyst, which is due to the interaction of the CeO_2_ support with the Cu nanoparticles. The Cu/CeO_2_ shows high selectivity to methanol due to the formation of carbonate intermediates, which are closely related to the oxygen vacancies on Cu/CeO_2_ (Figure 6). However, the low stability of CeO_2_ with excess of oxygen defects in the presence of large amounts of water limits its applicability.

Magnesium oxide (MgO) has also been explored as a support for the Cu particles because this support leads to the formation of small and highly dispersed metallic Cu particles with improved catalytic performance in methanol synthesis from CO_2_ [96]. The improvement in catalytic performance is also associated to the basicity of the MgO which facilitates the adsorption of CO_2_ and modifies the reaction pathways in its hydrogenation [45]. The methanol yield on Cu/MgO catalysts is favoured by the hydrogenation of the dioxomethylene intermediates and the moderate binding of the CO produced by RWGS [45]. The activity of the Cu/MgO catalysts prepared by co-precipitation depends on the hydroxycarbonate precursors which they derive, and, therefore, the control of the pH during the co-precipitation is a key factor [45]. In standard conditions, the precursor structure from the conventional co-precipitation is malachite which was observed in Cu/MgO precursors precipitated at acidic pH (3–7). The increase in the pH during co-precipitation leads to precursors with improved CuO dispersion, BET surface area and increase in the number of basic sites due to the formation of CuAl_2_O_4_ and MgAl_2_O_4_ spinel phases. These changes imply that the conversion of CO_2_ on the catalysts prepared at more basic pH was higher in comparison with the catalysts prepared at acidic pH, although the selectivity to methanol was lower [45].

Titanium oxide (TiO_2_) have also been explored as supports of Cu in the CO_2_ hydrogenation to produce methanol because the redox properties of the TiO_2_ facilitate the creation of oxygen vacancy sites which improve the CO_2_ activation. On the other hand, the high specific surface of TiO_2_ could also improve the reactivity and the dispersion of the active copper sites. The ability of TiO_2_ to create oxygen vacancies allows it to take the same role of ZnO in the mechanism of CO_2_ hydrogenation [91]. Therefore, the activation of CO_2_ could occur in the oxygen vacancies of the TiO_2_ support or in the interfacial sites between the Cu active metal and the oxide support [97].

### 4.4. Non-Cu Catalysts

#### 4.4.1. Noble Metals

Other active phases in addition to copper also have high reactivity for the selective hydrogenation of CO_2_ to methanol. These alternative active phases are mainly focused on Pd [47,98,99,100,101] and to a lesser extent on Au [102,103,104,105,106].

Palladium metal (Pd) is the second most studied active phase for methanol synthesis in the literature after the traditional Cu–ZnO system. Palladium is very active for the hydrogenation of CO_2_, and the selectivity to methanol depends on the type of support and promoters [98]. Palladium supported on ZnO leads to the formation of bimetallic PdZn alloy which acts as active phase for the selective production of methanol [98]. The bimetallic PdZn alloy phase is formed by reduction and, therefore, the reduction conditions determine the extent and characteristics of the formed PdZn alloy. In this sense, small PdZn particles (<4 nm) formed by reduction at 250 °C are highly active and selective for the production of methanol from CO_2_ (Table 5). The nature of the active sites in Pd/ZnO catalysts obtained after reduction (Pd^0^ and PdZn) defines the final selectivity in the hydrogenation of CO_2_, because the presence of metallic Pd nanoparticles only favours the formation of CO by the RWGS reaction, while the PdZn alloy particles are selective for the formation of methanol [98].

In a similar way, Pd supported on gallium oxide (Ga_2_O_3_) was also studied as active and selective catalysts for the synthesis of methanol from CO_2_ [98]. In this case, the interaction of Pd with Ga_2_O_3_ after reduction leads to the formation of not only the Pd–Ga alloy, but it also forms Pd–Ga intermetallic compounds. The Pd_2_Ga intermetallic compounds shows improved activity and methanol selectivity in respect to Pd–Ga alloy and conventional Cu–ZnO catalysts, because the intermetallic compounds provide atomic hydrogen to the surface of Ga_2_O_3_ that hinders both the decomposition of methanol and the production of CO [99]. The formation of Pd_2_Ga intermetallic compounds was also reported by Ota et al. [100] for catalysts obtained after reduction of substituted hydrotalcite precursors which show improved activity and methanol selectivity in the synthesis of methanol from CO_2_. Other types of palladium intermetallic compounds active in the synthesis of methanol from CO_2_ are those derived from the combination of Pd and In_2_O_3_ [101]. These PdIn intermetallic nanoparticles are found as highly efficient catalysts in the synthesis of methanol at 5.0 MPa, 210 °C and H_2_:CO_2_ = 3:1. The optimal catalyst consists of 8 nm nanoparticles comprising an intermetallic phase of PdIn enriched on the surface of In_2_O_3_. The PdIn-catalyst shows 70% higher methanol rates compared to the conventional Cu/ZnO/Al_2_O_3_ catalyst (900 and 540 μmol CH_3_OH (mmol (PdIn)^−1^ or (CuZnAl)^−1^) h^−1^, respectively). In addition, the same studies indicate the improvement in stability of PdIn catalysts respect to the conventional Cu/ZnO/Al_2_O_3_ catalysts. Thus, the rate of methanol production decreased by 20% after 120 h for the optimal PdIn phase compared to 30% for the conventional Cu/ZnO/Al_2_O_3_ catalyst (after 25 h). The analysis of used PdIn catalysts shows the same bimetallic phase of PdIn with only a slight increase in the size of the nanoparticles.

Gold in the form of nanoparticles has been reported as highly active catalyst for CO_2_ hydrogenation. Vourros et al. [103] carried out a complete experimental study about the activity of Au nanoparticles supported on several oxides to catalyse the CO_2_ hydrogenation to methanol at atmospheric pressure. Five different M_x_O_y_ oxide-supports were studied: Al_2_O_3_, TiO_2_, Fe_2_O_3_, CeO_2_, and ZnO. The gold nanoparticles supported on ZnO and CeO_2_ were highly selective towards methanol (nearly 90%, and 82% respectively at temperature below 250 °C). Hartadi et al. [104] also carried out a systematic study with different supports for Au nanoparticles and also found the best behaviour for the synthesis of methanol when supported on ZnO. The behaviour of Au/ZnO based catalysts was compared with that of the conventional Cu-Zn-Al catalyst (at 0.5–5.0 MPa and 240 °C [105]) and it was found that the Au/ZnO catalysts have similar methanol formation rate but higher selectivity towards methanol compared to conventional Cu/ZnO/Al_2_O_3._ The mechanistic studies of the hydrogenation of CO_2_ on Au/ZnO catalysts carried out from kinetic data and in situ infrared spectroscopy (IR) measurements concluded that the formation of methanol from CO_2_ and CO proceeds through different independent reaction pathways and CO was not an intermediate in the hydrogenation of CO_2_ over Au/ZnO catalysts. The Au nanoparticles supported on TiO_2_ and Fe_2_O_3_ showed a high conversion of CO_2_ (40% and 27%, respectively) which leads, however, almost exclusively to CO and/or CH_4_. The comparative study of CeO_2_ and TiO_2_, two reducible supports that allow the creation of oxygen vacancies, showed that the TiO_2_ support is more active (higher CO_2_ conversion), although the CeO_2_ support is more selective to methanol. Gold nanoparticles supported on Al_2_O_3_ catalyst were practically inactive in the conversion of CO_2_ to methanol in the investigated pressure (atmospheric) and temperature range (200–350 °C). Zirconium oxide (ZrO_2_) was also studied as a support for sub-nanometer particles of Au [105] which are able to produce methanol at low temperature (at 180 °C, the TOF and selectivity to methanol can reach 20 mol CH_3_OH/mol Au and 73 %, respectively).

Bimetallic alloys of noble metals (Rh and Pt) have been also investigated as alternative phases to Cu–ZnO for the synthesis of methanol from CO_2_. In this sense, the RhW alloy with nanosheet structure has been described as a catalyst for methanol synthesis with high activity and selectivity to methanol [107]. Compared with nanoparticles of monometallic Rh catalysts, the nanosheets of RhW alloy improve the adsorption and activation of CO_2_ due to the fact of the higher d-band level induced by one-dimensional quantum confinement effect and the negatively charged surface of Rh derived from the electronic effects associated with the alloy formation. Likewise, Bai et al. [108] reported the high activity and selectivity for methanol synthesis of catalysts based on PtCo alloys. In special catalysts formed by zigzag nanowires of PtCo alloy with Pt-rich surfaces and abundant steps/edges (defects) are reported as highly active and stable CO_2_ hydrogenation catalysts. Among the PtCo alloys, the Pt_4_Co was the stoichiometry most active for the synthesis of methanol from CO_2_. The result of infrared spectroscopy of CO_2_ adsorption shows that this nanowire Pt_4_Co alloy facilitates the adsorption/activation of CO_2_ through the formation of carboxylate intermediates and, therefore, improves methanol production.

#### 4.4.2. Non-Noble Metals and Oxides

Transition metals (Cu, Co, and Fe) supported on Mo_2_C was reported by Chen et al. [109] as active catalysts for the selective hydrogenation of CO_2_ to methanol under mild conditions (135–200 °C in a liquid solvent of 1,4-dioxane). The Mo_2_C served as a support and co-catalyst for the hydrogenation of CO_2_. Using pure Mo_2_C, methanol was the main product at 135 °C, while hydrocarbons, methanol, ethanol, and C^2+^ compounds were produced at 200 °C. Only in the case of the addition of Cu to the Mo_2_C improved the production of methanol, while in the case of the addition of Co and Fe they only increased the production of C^2+^ hydrocarbons [109].

Small particles of Ni*_x_*In*_y_*Al (*x* = 0–8.3, *y* = 0–9.1) with an average diameter from 2.5 to 3.5 nm supported on SiO_2_ were also reported as active catalysts for the synthesis of methanol from CO_2_ and H_2_ at low pressure [110]. The Ni*_x_*In*_y_*Al catalyst with Ni/In ratios of 0.4–0.7 were the formulations with the highest intrinsic activity (0.33 mol CH_3_OH (mol_metal catalyst_)^−1^ h^−1^). This formulation shows much higher activity than those obtained with the conventional Cu/ZnO/Al_2_O_3_ catalyst (0.17 mol CH_3_OH (mol_metal catalyst_)^−1^ h^−1^).

**Figure 7:**
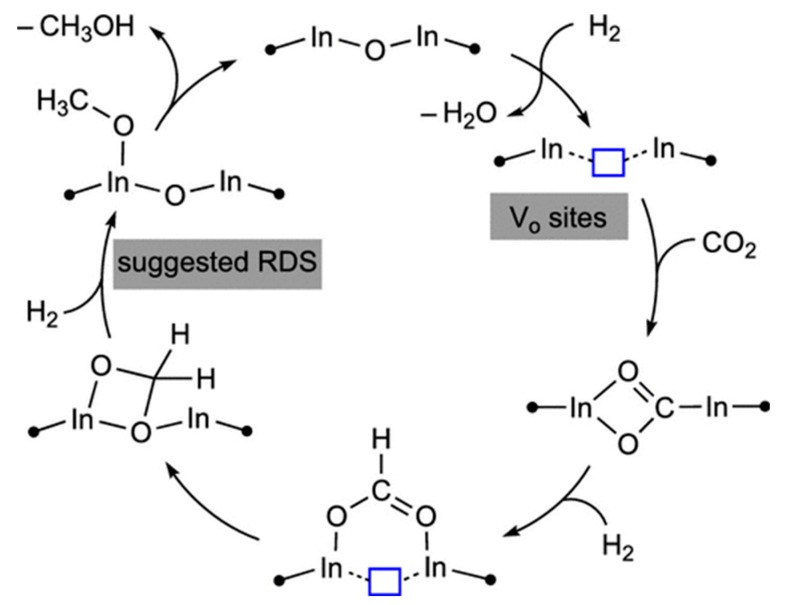
Site (oxygen vacancy) for methanol synthesis from CO_2_ hydrogenation on In_2_O_3_ (110). Reprinted with permission from Reference 112). Copyright (2019) American Chemical Society.

The oxides of indium, In_2_O_3,_ have attracted much interest in recent years as catalysts for methanol synthesis due to the fact of its capacity to create oxygen vacancies on its surface [53]. This catalyst allows for reaching methanol yields of 3690 μmol CH_3_OH g_cat_^−1^ h^−1^ [111]. Theoretical studies with density functional calculations (DFT) calculations suggest that the hydrogenation reaction of CO_2_ on the surface of In_2_O_3_ follows a mechanism consisting of the cyclic creation and the elimination of oxygen vacancies, as illustrated in Figure 7 [112]. These mechanistic studies seem to indicate that the mechanism of methanol formation on a defective oxygen centre of In_2_O_3_ is different from the mechanism operating in the conventional Cu-based catalysts. This is because the hydrogenation of CO_2_ to HCOO is thermodynamically and kinetically favourable over the oxygen vacancies on the surface of In_2_O_3_, which can also stabilize the key intermediates involved in the formation of methanol, including HCOO, H_2_COO and H_2_CO. On the contrary, the H_2_CO and H_2_COO species are not stable on the surface of Cu (111) [53,111]. 

The Indium oxide (In_2_O_3_) has been combined with ZrO_2_, obtaining composites [113] with high selectivity to methanol (100%) even at a high temperature (T > 300 °C). Moreover, this In_2_O_3_–ZrO_2_ composite shows high stability for 1000 h under industrially relevant conditions (P = 5.0 MPa, H_2_:CO_2_ = 4:1, and GHSV = 16,000 h^−1^). These contrast strongly with conventional Cu/ZnO/Al_2_O_3_ catalysts, which are non-selective and undergo rapid deactivation. The in-depth characterization of the In_2_O_3_/ZrO_2_ composite points to a mechanism rooted in the creation and elimination of oxygen vacancies as active sites, whose quantity can be modulated in situ by the co-feeding CO and through electronic interactions with the zirconia carrier [113].

In a similar way Akkharaphatthawona et al. [114] developed new In_2_O_3_–Ga_2_O_3_ composites (Ga*_x_*In_2-*x*_O_3_) highly active for the selective hydrogenation of CO_2_ to methanol at high temperature (320–400 °C). The phase, crystallinity, porosity, morphology and the electronic properties of the Ga*_x_*In_2-*x*_O_3_ composites can be modified by adjusting their Ga/In ratio, and these properties determine their reactivity in the hydrogenation of CO_2_. At the lowest temperature studied (320 °C), pure In_2_O_3_ shows the highest methanol yield. However, the methanol yield decreases significantly with increasing reaction temperatures. The incorporation of Ga into the crystalline lattice of In_2_O_3_ at x = 0.4 (Ga_0.4_In_1.6_O_3_) maximizes the methanol yield at higher reaction temperature (340–360 °C). This improvement can be attributed to an increase in the binding energy of the adsorbed intermediate molecules on the surface of the catalyst which promotes the selective hydrogenation of CO_2_ to methanol. A higher content of Ga in the Ga*_x_*In_2-*x*_·O_3_ composites (x > 0.4) leads to a great strengthening of the adsorbed intermediate molecules, resulting in a lower methanol yield and methane formation.

## 5. Conclusions and Prospects

The development of competitive catalytic technologies for the selective catalytic hydrogenation of CO_2_ to methanol offers a path forward into a carbon neutral society reducing the huge CO_2_ emissions from fossil fuels by converting them into fuels and chemicals. Since the early 1990s, the chemical valorisation of CO_2_ by hydrogenation to methanol has been the focus of research and much effort has been spent in the development of catalysts. For the wider adoption of this technology, important advances must be made in both catalysts with the specific needs of the reaction conditions and process design and with reactors and separators adjusted to plants of small capacity to minimize the gas recycle volume. As shown in preceding sections, preparation, composition and nano-structural characteristics of catalysts are the core for the selective catalytic hydrogenation of CO_2_ to methanol. Catalysts for methanol synthesis from CO_2_ hydrogenation based on conventional Cu/ZnO will continue to be the most widely used formulation for CO_2_-to-methanol production because of its optimal activity/cost ratio. However, Cu/ZnO catalysts are not optimized for CO_2_-to-methanol and challenges, such as the improvement in its low temperature activity, time-on-stream deactivation behaviour and formation of by-products, need to be solved. The Cu–ZnO catalyst has been studied for the synthesis of methanol from syngas or CO_2_ for decades; however, we still do not know in detail the key aspects which determine its activity/selectivity: the genesis and the exact nature of the active site and the reaction mechanism under real reaction conditions. Deep studies on synthesis approaches for Cu–ZnO catalysts should be developed, since they control, to a large extent, the catalyst performance. Therefore, the understanding of the evolution of the interfacial and local properties of Cu–ZnO catalysts that greatly determine its activity/selectivity from the precursors to the catalysts under reaction conditions is needed. The final elucidation of the surface composition and structure of the active sites of Cu–ZnO catalysts under working conditions using advanced *operando* spectroscopic techniques is highly desirable. In addition to experimental characterization, theoretical and modelling work is also necessary for a true understanding of the process and mechanisms operating in the selective hydrogenation of CO_2_ to methanol. The determination of the reaction mechanisms at a molecular level will help to provide a real picture of the different reaction pathways on catalytic surfaces, which may allow us to design and maximize the number of catalytic active/selective sites for methanol synthesis on the catalytic surfaces under working conditions. In most cases, activity and durability of Cu–ZnO catalysts can be increased dramatically by incorporating promoters. Hence, appropriate knowledge about promoting mechanisms is important in order to host the promoter elements in well-defined and tailored locations. In addition to the development of more active and selective catalysts, the deactivation of the catalysts should not be forgotten. Phenomena affecting the stability of catalysts should also be characterized in order to determine the main causes under working conditions that affect the destruction of active sites to ensure the maximum longevity of catalyst.

Finally, novel catalysts are expected to be developed, while the Cu–ZnO should be optimized. The accumulation of a large body of experimental and theoretical work is important in the search for new catalysts and their optimization for methanol synthesis from CO_2_. Databases including information regarding the syntheses, active phases, promoters, activity and stability, among others, are useful to avoid repeated, unnecessary work and to critically evaluate the practical viability of alternative catalyst formulations. Among the alternative formulations to the Cu–ZnO systems, those based on Pd, Au, and In_2_O_3_ have sufficient potential to overcome the constraints observed in the conventional Cu–ZnO catalysts, but they still need improvements to meet the requirements that the industrial application of CO_2_ hydrogenation to methanol needs.

## Figures and Tables

**Table 1:** Main chemicals products industrially produced from CO_2_ [5].

Chemical	Molecular Formula	Production (t/year)	CO_2_ Consumption (t/year)
Urea	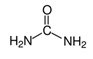	1.5 × 10^8^	1.12 × 10^8^
Methanol	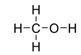	1.0 × 10^8^	2 × 10^6^
Formaldehyde	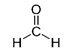	9.7 × 10^6^	
Formic acid	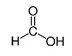	7.0× 10^5^	
Salicylic acid	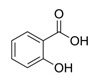	7.0 × 10^4^	3.0 × 10^4^
Cyclic carbamate	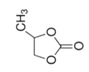	8.0 × 10^4^	4.0 × 10^4^
Ethylene carbamate	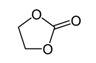		
Di-methyl carbamate	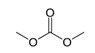	1.0 × 10^7^	
Copolymers	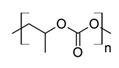		
Polymer-building blocks			
Fine chemical: for example, biotin	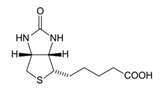		

**Table 2:** Effect of Zr on the activity and selectivity for methanol synthesis on Cu–Zn–Al–Zr catalysts (523 K and 5 MPa) [76].

Sample	Atomic Ratio^a^ Cu^2+^:Zn^2+^:Al^3+^:Zr^4+^	CO_2_ Conversion (%)	CH_3_OH Yield (μmol mL^−1^ h^−1^)	Selectivity (C mol%)	
				CH_3_OH	CO
Cal.HTs-0	2:1:1:0	20.2	3433	42.3	57.7
Cal.HTs-1	2:1:0.9:0.1	21.9	4057	45.8	54.2
Cal.HTs-3	2:1:0.7:0.3	22.5	4369	47.4	52.6
Cal.HTs-5	2:1:0.5:0.5	19.5	3745	44.0	56.0
Cal.HTs-7	2:1:0.3:0.7	15.3	2497	37.1	62.9

^a^ Nominal atomic ratio in the synthesis mixture.

**Table 3:** Effect of Ga on the catalytic activity and stability in CH_3_OH synthesis over Cu–Zn–Al–Ga catalysts (523 K and 3.0 MPa) [66].

Sample	Nominal Composition (at.%)				CO_x_ Conversion (%)	CH_3_OH Yield (mmol g_cat_^−1^ min^−1^)
	Cu	Zn	Ga	Al		
CZ-0.00	70	30	0.0	0.0	11.48	688
CZA-0.00	68	29	0.0	3.0	13.70	812
CZAG-0.33	68	29	1.0	2.0	15.14	906
CZAG-0.50	68	29	1.5	1.5	14.95	897
CZG-1.00	68	29	3.0	0.0	13.38	814

**Table 4:** Effect of modification of Cu–ZnO catalysts with ZrO_2_, CeO_2_ and Al_2_O_3_ over the activity for methanol synthesis (553 K and 5.0 MPa) [81].

Sample	M^a^ (wt.%)			CO_x_ Conversion (%)	CH_3_OH Yield (mmol g_cat_^−1^ h^−1^)	CH_3_OH Selectivity (%)
	Al	Zr	Ce			
CZ-Al_2_O_3_	100	-	-	19.5	9707	37
CZ-CeO_2_	-	-	100	12.8	6554	37
CZ-ZrO_2_	-	100	-	23.2	10,331	33

^a^ Nominal composition (wt.%) Cu/Zn/Me (Me: Al, Ce,s and/or Zr) = 30/41/29.

**Table 5:** Pd/ZnO prepared by impregnation (IM) and sol immobilization (SI) methods. Effect of the reduction pre-treatment on the conversion and selectivity for methanol synthesis from CO_2_ [98].

Sample	H_2_ Pre-Treatment	CO_2_ Conv. (%)	CH_3_OH Yield (μmol g_cat_^−1^ h^−1^)	Selectivities (%)	
	PdZn Alloy Formation *			CH_3_OH	CO
5% Pd/ZnO IM	150 °C	0	0	0	0
	250 °C	4.5	0	0	100
	400 °C	6.7	52	2	98
	550 °C	9.5	0	0	100
	700 °C	0.7	69	26	74
5% Pd/ZnO SI	150 °C	8.7	1900	48	52
	250 °C	7.9	2100	58	42
	400 °C	10.7	2423	60	39
	550 °C	6.3	1700	64	36
	700 °C	5.6	1400	72	28

Reaction conditions: 0.5 g catalyst, 30 mL/min of H_2_:CO_2_ = 3:1 mixture, 2.0 MPa, 250 °C, time 3 h. * XPS and XRD analyses: at 150 °C Pd is as metal Pd^0^. The extent of formation of PdZn alloy increases upon increasing reduction temperature.
